# Proteomic and Phosphoproteomic Signatures Link Molecular Remodeling to Behavioral Outcomes Following Elderberry and DHA Supplementation in Aging Mice

**DOI:** 10.3390/nu18172802

**Published:** 2026-08-27

**Authors:** Wenyan Yu, Jiankun Cui, Tamanna Mony, Stephen Mensah, Marcus Jackson, Runting Li, Ethan Pan, Grace Y. Sun, Andrew L. Thomas, C. Michael Greenlief, Zezong Gu

**Affiliations:** 1 Department of Pathology and Anatomical Sciences, University of Missouri School of Medicine, Columbia, MO 65212, USA; wyxxd@missouri.edu (W.Y.); cuij@missouri.edu (J.C.); tmhb8@missouri.edu (T.M.); mnjdgx@health.missouri.edu (M.J.); lirun@missouri.edu (R.L.); ep3h8@missouri.edu (E.P.); sung@missouri.edu (G.Y.S.); 2 Department of Chemistry, University of Missouri, Columbia, MO 65211, USA; smfrb@missouri.edu (S.M.); greenliefm@missouri.edu (C.M.G.); 3 Department of Biochemistry, University of Missouri, Columbia, MO 65211, USA; 4 Division of Plant Science and Technology, University of Missouri Southwest Research, Extension, and Education Center, Mount Vernon, MO 65712, USA; thomasal@missouri.edu

**Keywords:** elderberry, DHA, dietary supplementation, aging, proteomics, phosphoproteomics, fatty acids, synaptic plasticity, behavior, *Sambucus*, lipid metabolism, spatial memory, anxiety-like behavior, Ingenuity Pathway Analysis

## Abstract

**Background**: Aging is a risk factor for Alzheimer’s disease and related dementias, which are associated with synaptic dysfunction and cognitive decline. Elderberry (*Sambucus* spp.) is rich in anthocyanins with antioxidant and anti-inflammatory properties. Docosahexaenoic acid (DHA), an essential fatty acid, plays a key role in neuronal membrane integrity during brain aging. However, it remains unclear whether elderberry and DHA exert overlapping or distinct effects on brain aging and how these relate to molecular signaling. This study aimed to characterize molecular signatures induced by dietary supplementation and to determine their relationships with behavioral outcomes. **Methods**: 44-week-old male C57BL/6J mice were randomly assigned to control, elderberry, DHA, or combined diets for 12 weeks. Behavioral testing assessed anxiety-like behavior, spatial learning and memory. Brain tissues underwent proteomic and phosphoproteomic profiling and fatty-acid analysis. Data were analyzed using Ingenuity Pathway Analysis to identify enriched pathways, upstream regulators, and functional associations. **Results**: Elderberry as well as DHA supplementation induced targeted remodeling of the proteome and phosphoproteome, with pathway enrichment involving synaptogenesis, glutamatergic signaling, and long-term potentiation. Upstream-regulator analysis predicted elderberry-associated CDK5 signaling, accompanied by reduced MAPT/Tau phosphorylation at selected sites, whereas DHA supplementation was associated with CAMK-related signaling. DHA supplementation altered fatty-acid composition, increasing the *n*-3/*n*-6 ratio. Elderberry reduced anxiety-like behavior and improved target-directed search during the Barnes maze probe test. Molecular signatures were examined in relation to the measured behavioral outcomes. **Conclusions**: Elderberry and DHA are associated with distinct molecular networks related to synaptic function and behavioral outcomes in the aging male mouse brain. These findings support further investigation of elderberry and DHA as dietary interventions targeting molecular and behavioral features of brain aging.

## 1. Introduction

Aging is characterized by progressive decline in physiological function and represents the primary risk factor for Alzheimer’s Disease and Related Dementias (ADRD) [1,2]. With the global prevalence of dementia projected to exceed 153 million by 2050, there is an urgent need to develop non-invasive strategies to preserve cognitive function [2,3]. The aging brain is increasingly susceptible to oxidative stress and chronic low-grade inflammation, contributing to synaptic dysfunction and cognitive decline [4]. This age-associated environment disrupts redox homeostasis and impairs antioxidant defenses in the aging brain [5]. These changes manifest behaviorally as impaired spatial memory, reduced exploratory activity, and increased anxiety-like behavior in murine models [6].

Elderberry (*Sambucus* spp.) has gained attention as a potential nutraceutical for mitigating age-related neurological decline due to its high anthocyanin content, particularly cyanidin glycosides, which are present at relatively high concentrations compared with many other berries. Elderberry-derived preparations vary according to the plant part, species, cultivar, and processing method, and fruit-derived products should not be considered phytochemically equivalent to flower preparations [7,8]. In the present study, we used a spray-dried juice powder produced from the ripe fruit of American elderberry, *Sambucus nigra* subsp. *canadensis*, cultivar ‘Bob Gordon’, following a previously described procedure [9,10]. Elderberry anthocyanins and other phenolic constituents exhibit antioxidant and anti-inflammatory properties, including modulation of redox-sensitive and inflammatory signaling pathways, that may promote molecular homeostasis [11,12,13,14,15,16,17,18]. More broadly, flavonoids and anthocyanins can modulate redox-sensitive, inflammatory, and apoptotic pathways relevant to brain aging and neurodegeneration [19,20,21,22]. Although elderberry has been widely studied for its systemic effects, growing evidence supports its potential relevance to brain health. *In vitro* studies have shown that elderberry extracts and selected phenolic constituents can attenuate microglial activation [13]. In addition, dietary supplementation containing elderberry attenuated cerebral ischemia-related neuronal damage in a mouse model [14]. Clinical studies using American elderberry juice suggest potential cognitive benefits, including improvements in cognitive flexibility in individuals with mild cognitive impairment [23,24]. Anthocyanins have also been shown to modulate synaptic plasticity-related processes relevant to learning and memory. However, the effects of long-term elderberry intake on the molecular landscape of the aging brain, particularly with respect to synaptic protein regulation and signaling pathways, remain unclear.

Docosahexaenoic acid (DHA), a long-chain omega-3 polyunsaturated fatty acid, plays a critical role in neuronal membrane structure, synaptic function, and neuroinflammatory balance [25,26,27,28]. DHA can influence membrane organization, signal transduction, neurotransmission, and synaptic plasticity, thereby contributing to neural function and brain health throughout the lifespan [29,30,31,32]. Age-related reductions in brain DHA may impair synaptic plasticity and increase neuronal vulnerability to oxidative stress and inflammation [33]. Prior studies and reviews have examined the relationship of dietary DHA or long-chain omega-3 fatty-acid intake and cognitive function during aging [34,35,36,37,38]. Additional evidence indicates that DHA may exert neuroprotective effects through membrane- and metabolite-mediated signaling and by modulating glutamate-related excitotoxicity, oxidative stress, inflammation, and neuronal survival [39,40]. Although elderberry polyphenols and DHA represent distinct classes of dietary bioactives, both may influence key features of brain aging, including oxidative stress, inflammation, and synaptic dysfunction. Elderberry primarily provides antioxidant and anti-inflammatory effects through redox-sensitive signaling pathways, whereas DHA contributes to membrane integrity and lipid-mediated signaling pathways. However, whether these interventions are associated with shared or distinct molecular responses remains unclear, particularly given that dietary supplements are often consumed in combination. Therefore, it is important to determine whether elderberry, DHA, and their combined supplementation produce shared or distinct molecular and behavioral response patterns in the aging brain. Most studies on elderberry have focused on systemic antioxidant markers, inflammation, or behavioral outcomes, while protein networks remain underexplored [15,23]. DHA has been extensively studied for its roles in brain-lipid metabolism and neuroinflammation [25,26,27,33,41]. Emerging evidence also suggests that DHA is associated with changes in protein networks involved in inflammatory and oxidative processes. However, it remains unclear whether elderberry, DHA, and their combined supplementation alter protein expression and phosphorylation-dependent signaling in the aging brain. Cognitive functions, such as spatial learning and memory, depend on coordinated molecular processes, including synaptogenesis, glutamatergic neurotransmission, and long-term potentiation [42]. Global proteomic profiling enables systematic identification of protein-level changes induced by dietary interventions, while phosphoproteomic analysis provides insight into dynamic signaling regulation through protein phosphorylation [43]. Recent proteomic and phosphoproteomic studies of the aging mouse brain and liver have identified age-related changes in protein abundance, phosphorylation sites, and associated molecular pathways [44,45]. Therefore, integrated analysis of the proteome and phosphoproteome is needed to identify molecular signatures associated with elderberry, DHA, and their combination, and to determine how these molecular changes relate to cognitive function during aging.

In this study, we investigated the effects of a 12-week dietary intervention with elderberry, DHA, and their combination in aging mice. We combined behavioral assessment with proteomic, phosphoproteomic, and fatty acids composition analyses to identify molecular networks correlated to functional outcomes. This integrative approach provides insight into molecular signaling networks associated with dietary supplementation in the aging brain.

## 2. Materials and Methods

### 2.1. Animals

A total of 47 male C57BL/6J mice (RRID:IMSR_JAX:000664) were obtained from The Jackson Laboratory (Bar Harbor, ME, USA). Mice were housed under a reversed 12-h light/dark cycle with *ad libitum* access to food and water. All experimental procedures were reviewed and approved by the Institutional Animal Care and Use Committee (IACUC) at University of Missouri (Protocol no. 25210 approved on 10 September 2021). Animals received routine husbandry and veterinary care and were observed daily by animal-facility personnel for general health and signs of pain or distress. No diet-related adverse events were observed.

### 2.2. Diet/Supplement

The elderberry preparation consisted of spray-dried juice powder produced from the ripe fruit of American elderberry (*Sambucus nigra* subsp. *canadensis*), cultivar ‘Bob Gordon’. The berries were harvested by Elder Farms (Mt. Vernon, MO, USA), commercially de-stemmed, sorted, and promptly frozen. The berries were later thawed and pressed to produce juice, with the resulting juice spray-dried using soy protein isolate as the carrier following a previously described procedure [10]. Published characterization of the corresponding elderberry juice–soy protein isolate formulation reported 3091.88 ± 43.07 mg total anthocyanins per 100 g powder and approximately 32 mg gallic acid equivalents per gram powder of total polyphenols [10].

The DHA source was algae oil derived from *Schizochytrium* sp. containing 44.3% DHA (Jedwards International, Braintree, MA, USA). Approximately 2.3% (*w*/*w*) algae oil was incorporated into AIN-93G to provide a final DHA concentration of approximately 1% (*w*/*w*).

At 44 weeks of age, equivalent to middle age in human [46], housing cages were randomly assigned to four dietary groups, with all mice within each cage receiving the same diet for 12 weeks: control diet (*n* = 12, AIN-93G; Product# F3156—Rodent Diet, Bio Serv^®^, Flemington, NJ, USA); elderberry (*n* = 11); DHA (*n* = 12); combined elderberry + DHA (*n* = 12). The cage was the experimental unit for dietary allocation and food-intake measurements, whereas the individual mouse was the experimental unit for body weight, behavioral, proteomic, phosphoproteomic, and fatty-acid analyses. The elderberry diet contained 2% elderberry juice powder, the DHA diet contained approximately 1% (*w*/*w*) DHA, and the combined diet contained both 2% elderberry juice powder and approximately 1% DHA. Investigators conducting behavioral testing and outcome assessment were blinded to dietary-group allocation.

Estimated daily mouse exposure was calculated from the dietary concentration and cage-level food intake normalized to body weight. Human-equivalent exposure was estimated using body-surface-area scaling, with K_m_ values of 3 for mice and 37 for adult humans [47] and provided only as approximate translational comparison.

### 2.3. Behavioral Testing Conditions

All behavioral experiments were conducted during the dark phase under a reversed light/dark cycle. Mice were transferred to the testing room and allowed to acclimate for at least 1 h before the start of testing. This acclimation period minimized stress and ensured stable behavioral performance across assays.

### 2.4. Elevated Plus Maze (EPM)

Anxiety-like behavior was assessed using the elevated plus maze (EPM; Stoelting company, Wood Dale, IL, USA) based on the protocol described by Lister [48]. A total of 47 mice were included: control (*n* = 12); elderberry (*n* = 11); DHA (*n* = 12); combined (*n* = 12). This test exploited rodents’ natural avoidance of open and elevated spaces and preference for enclosed areas. Accordingly, increased time spent in the closed arms was interpreted as greater anxiety-like behavior. The maze consisted of a Plexiglas apparatus with a central area (5 × 5 cm) and four arms (5 × 45 cm). Two opposite arms were enclosed by 15 cm high walls (closed arms), while the other two arms were open (open arms). The maze was elevated 45 cm above the floor.

At the start of each trial, each mouse was placed on the central area facing a closed arm and allowed to explore the maze freely for 5 min. Behavior was recorded using a video camera and analyzed with an ANY-maze automated video-tracking system (RRID:SCR_014289; version 7.42; Stoelting Co., Ltd., Wood Dale, IL, USA). The time spent and the number of entries into the open arms, closed arms, and the central zone were quantified.

### 2.5. Open Field Test (OFT)

Locomotor activity, exploration behavior, and anxiety-like behavior were assessed using the open-field test (Maze Engineers, Skokie, IL, USA), as previously described [49,50]. A total of 46 mice were included: control (*n* = 12); elderberry (*n* = 11); DHA (*n* = 11); combined (*n* = 12). One mouse was excluded from the open-field test and emergence test analyses because repeated wall climbing resulted in unreliable automated tracking. The apparatus consisted of a 40 × 40 cm square platform. Each mouse was placed individually in the center of the arena and allowed to explore freely for 10 min. Animal movement was recorded using a video tracking system and analyzed with ANY-maze software. For spatial analysis, the arena was virtually divided into sixteen equal squares (10 × 10 cm). Four of the squares were defined as the center zone, while the remaining squares constituted the peripheral zone. Parameters including total distance traveled and time spent in the center versus the periphery were quantified as measures of locomotion and anxiety-like behavior.

### 2.6. Barnes Maze

Spatial learning and memory were assessed using the Barnes maze (San Diego Instruments, San Diego, CA, USA) as previously described [51,52,53,54]. A total of 34 mice were included: control (*n* = 9); elderberry (*n* = 7); DHA (*n* = 9); combined (*n* = 9). The maze consisted of a circular platform (75 cm in diameter) elevated to 56 cm above the floor, with 20 evenly spaced holes (5 cm in diameter) along the perimeter. One hole was designated as the escape hole with an escape box placed beneath it. The location of the escape hole remained constant for each mouse during the acquisition training.

To provide an aversive stimulus, the lights were positioned above the platform, encouraging mice to locate and enter the escape box. Four visual cues (+, ∎, ∆, •) were placed on surrounding curtains to facilitate navigation.

The test procedure included habituation, initial training, reversal training, and a probe test. On Day 1, mice underwent two habituation trials. Initial training was performed on Days 2–5. Reversal training was conducted on Days 6–8, during which the escape hole was relocated to the opposite position. Two trials per day were conducted with an inter-trial interval of 20–30 min. The maximum trial duration was 300 s. Performance during initial and reversal training was evaluated using overall task completion and latency among mice that completed at least one trial on each day of the corresponding training phase. A phase non-completer was defined as a mouse that failed to locate the escape hole within 300 s one or more days of the corresponding phase. Mice that failed to complete only one of the two trials on a given day were retained in the latency analysis, with the maximum latency of 300 s assigned for each unsuccessful trial. Mice whose automated-tracking records did not meet quality-control criteria were excluded from the corresponding latency analysis. On Day 9, a probe test was conducted with the escape box removed to evaluate spatial memory retention.

Latency to locate the escape hole and the number of errors (nose-pokes into non-escape holes) were recorded and analyzed using ANY-maze software. For the probe test, spatial memory retention was assessed based on search behavior around the target location.

### 2.7. Emergence Test

Novelty-induced anxiety and exploratory behavior were assessed using the emergence test, an open-field-based behavioral paradigm [55,56]. This test evaluates the conflict between the natural exploratory drive of mice and their avoidance of brightly lit, open environments. Increased latency to leave the shelter or prolonged time spent inside the shelter was interpreted as greater anxiety-like behavior. A total of 46 mice were included: control (*n* = 12); elderberry (*n* = 11); DHA (*n* = 11); combined (*n* = 12).

The test was conducted in the open-field arena, same as the OFT. A cylindrical tube (13.2 × 4.4 × 4.4 cm) was used as a temporary shelter and positioned in the center of the arena. At the start of each trial, the mouse was placed inside the tube, and the lid was removed to allow free exploration. Behavior was recorded and analyzed using ANY-maze software. Measured parameters included latency to emerge, time spent in the shelter, number of shelter entries, number of nose-pokes at the shelter opening, and time spent in the illuminated arena.

### 2.8. Data and Statistical Analysis for Behavior Tests

Data are presented as mean ± SEM. Statistical analyses were performed using one-way or two-way analysis of variance (ANOVA), as appropriate, with GraphPad Prism software (RRID:SCR_002798; version 11.0.1; GraphPad Software, LLC, San Diego, CA, USA). Dunnett’s or Tukey’s test was applied for multiple comparisons. Differences were considered statistically significant at *p* < 0.05.

### 2.9. Tissue Processing and Proteomic/Phosphoproteomic Analysis

A total of 20 mice were used for proteomic analysis, including control (*n* = 5), elderberry (*n* = 5), DHA (*n* = 5), and combined (*n* = 5) groups. After behavior tests, mice were deeply anesthetized by isoflurane overdose, and an adequate depth of anesthesia was confirmed by the absence of a pedal withdrawal reflex. Mice were then euthanized by decapitation in accordance with the approved IACUC protocol. The cortex and striatum were rapidly dissected for subsequent analyses. The cortex was processed for label-free global proteomic and phosphoproteomic analysis by tandem mass spectrometry (MS2). Briefly, tissues were homogenized in a denaturing lysis buffer containing 5% SDS, 50 mM Tris-HCl, and 10 mM DTT (six volumes, *w*/*v*) using a Fisherbrand Model 150 Homogenizer (Fisher Scientific, Waltham, MA, USA) on ice. Lysates were centrifuged at 17,000× *g* for 20 min at 4 °C, and the supernatant was collected. Proteins were reduced and alkylated with iodoacetamide in the dark and precipitated with four volumes (*w*/*v*) of cold acetone at −20 °C overnight. Protein pellets were washed twice with cold acetone and resuspended in urea/thiourea/ammonium bicarbonate buffer. Protein concentration was determined using the Pierce 660 nm Protein Assay kit (Thermo Fisher Scientific, Waltham, MA, USA) according to the manufacturer’s instructions.

Proteins were digested with trypsin using a sequential digestion protocol at a 1:100 enzyme-to-protein ratio and digestion was terminated with 0.5% trifluoroacetic acid. For global proteomic profiling, 500 ng of peptides from each sample were analyzed using long-gradient DIA-PASEF on an Evosep One liquid chromatography system coupled to a Bruker timsTOF Pro 2 mass spectrometer (RRID:SCR_026545; Bruker, Bremen, Germany). For phosphoproteomic analysis, the remaining peptides were desalted and enriched using the Fe-NTA Phosphopeptide Enrichment Kit (Thermo Fisher Scientific, Waltham, MA, USA) prior to LC-MS/MS analysis on the same DIA-PASEF platform. The phosphoproteomic workflow was performed as previously reported [57].

### 2.10. Mass Spectrometry Data Processing and Bioinformatic Analysis

Raw DIA-PASEF data were analyzed using the directDIA workflow in Spectronaut (version 19.7) with default settings. False identifications were controlled at a 1% false discovery rate at the peptide-spectrum match, peptide, and protein-group levels. Trypsin was used as the digestion enzyme with up to two missed cleavages allowed.

Carbamidomethylation of cysteine was set as a fixed modification, while methionine oxidation and protein *N*-terminal acetylation were set as variable modifications. For phosphoproteomic analysis, phosphorylation of serine, threonine, and tyrosine residues was included as an additional variable modification.

Proteomic and phosphoproteomic datasets were exported to Perseus (RRID:SCR_015753) for further processing and normalized by sample median. For phosphoproteomic data, peptide-level output from Spectronaut were converted to phosphosite-level tables using Peptide Collapse plugin (version 1.4.4), with a PTM assay probability cutoff of 0.75 for class I phosphorylation sites.

Differential protein abundance was assessed by calculating expression ratios and *p* value relative to control samples. Proteins and phosphoproteins meeting the *p* < 0.05 were classified based on the direction of change and retained for descriptive and pathway-prediction analyses. Global proteomic findings were used for descriptive comparison of protein-abundance patterns among dietary groups. For visualization, data were transformed to log_2_ fold change (FC) and −log10 *p* values, respectively.

The phosphoproteomic dataset was analyzed using Ingenuity Pathway Analysis (IPA; RRID:SCR_008653; Redwood City, CA, USA) to identify canonical pathways, upstream regulators, and functional associations, including behavior-related functions. For chord-diagram visualization using SRplot [58], a more exclusive display cutoff of |log_2_(FC)| ≥ 0.2 was applied to retain sufficient pathway-associated phosphoproteins for visualization of the coordinated phosphoprotein–pathway relationships. Functional predictions were compared with observed behavioral outcomes.

GO functional-enrichment analysis was performed separately for proteins with nominal *p* < 0.05 in the elderberry, DHA, and combined groups relative to control. Proteins with increased and decreased abundance were analyzed together using Metascape with Mus musculus annotations [59]. Enrichment *p* values were calculated using the cumulative hypergeometric distribution, and FDR-adjusted q values were calculated using the Benjamini–Hochberg procedure.

### 2.11. Brain Fatty Acid Composition Analysis by GC-FAME

Fatty acid methyl esters (FAMEs) standards, acetyl chloride, benzene, sodium sulfate anhydrous, and heptane were obtained from Sigma Millipore (MilliporeSigma, Burlington, MA, USA). Fatty-acids analyses in the left striatum tissue from the four dietary groups (*n* = 5 per group) were carried out by direct transesterification without prior lipid extractions, as described by Lepage and Roy [60]. Each biological sample was analyzed in technical triplicate, and the mean of the technical replicates was used as the value for statistical analysis. Briefly, samples were homogenized in eightfold volumes of HPLC water using a bullet blender (Next Advance, Inc., Averill Park, NY, USA). Tissue homogenate (150 μL) was transferred to a round bottom flask and dried under a stream of nitrogen. Samples were then mixed with 1 mL of methanol-benzene (3:2, *v*/*v*) and spiked with 50 μL of internal standard (C17:0 methyl ester, 1 mg/mL). Subsequently, 1 mL of freshly prepared acetyl chloride-methanol (5:100, *v*/*v*) was added, and methanolysis was performed at 100 °C for 1 h under reflux. After cooling to room temperature, 1 mL of HPLC grade water and 1 mL of hexane were added, followed by vigorous mixing. The upper hexane layer was collected, passed through anhydrous sodium sulfate, and evaporated under nitrogen. The residue was resuspended in 100 μL of heptane, and 1 μL was injected into the gas chromatograph. FAMEs were analyzed using an Agilent 8860 GC gas chromatograph system equipped with a DB-FastFAME column (30 m, 0.25 mm I.D., 0.25 μm film thickness; Agilent Technologies, Santa Clara, CA, USA). Helium was used as the carrier gas at a flow rate of 0.9 mL/min. The oven temperature was programmed from 80 °C (held for 0.5 min) to 230 °C (at 40 °C/min) and held for 4 min. The system operated at a constant pressure of 12 psi. FAs were identified by comparison with commercial standards. Results are expressed as the percentage of total FAs (g/100 g of tissues).

Data are presented as mean ± SEM. Statistical analyses were performed using one-way analysis of variance (ANOVA) with GraphPad Prism Software. Tukey’s post hoc test was applied for multiple comparisons. Differences were considered statistically significant at *p* < 0.05. For comparisons between two groups, an unpaired *t*-test was applied.

## 3. Results

### 3.1. Effects of Dietary Supplementation on General Health of Aging Mice and Physiological Parameters

No significant differences in average food intake or body weight were observed among the dietary groups (Figure S1). After an initial diet adaptation period (2 weeks), all groups maintained stable and comparable body weight profiles. No abnormalities in general health or unusual behaviors were observed outside of formal behavioral testing during routine monitoring.

### 3.2. Global Proteomic Profiling Identifies Differentially Expressed Proteins (DEPs)

**Figure 1:**
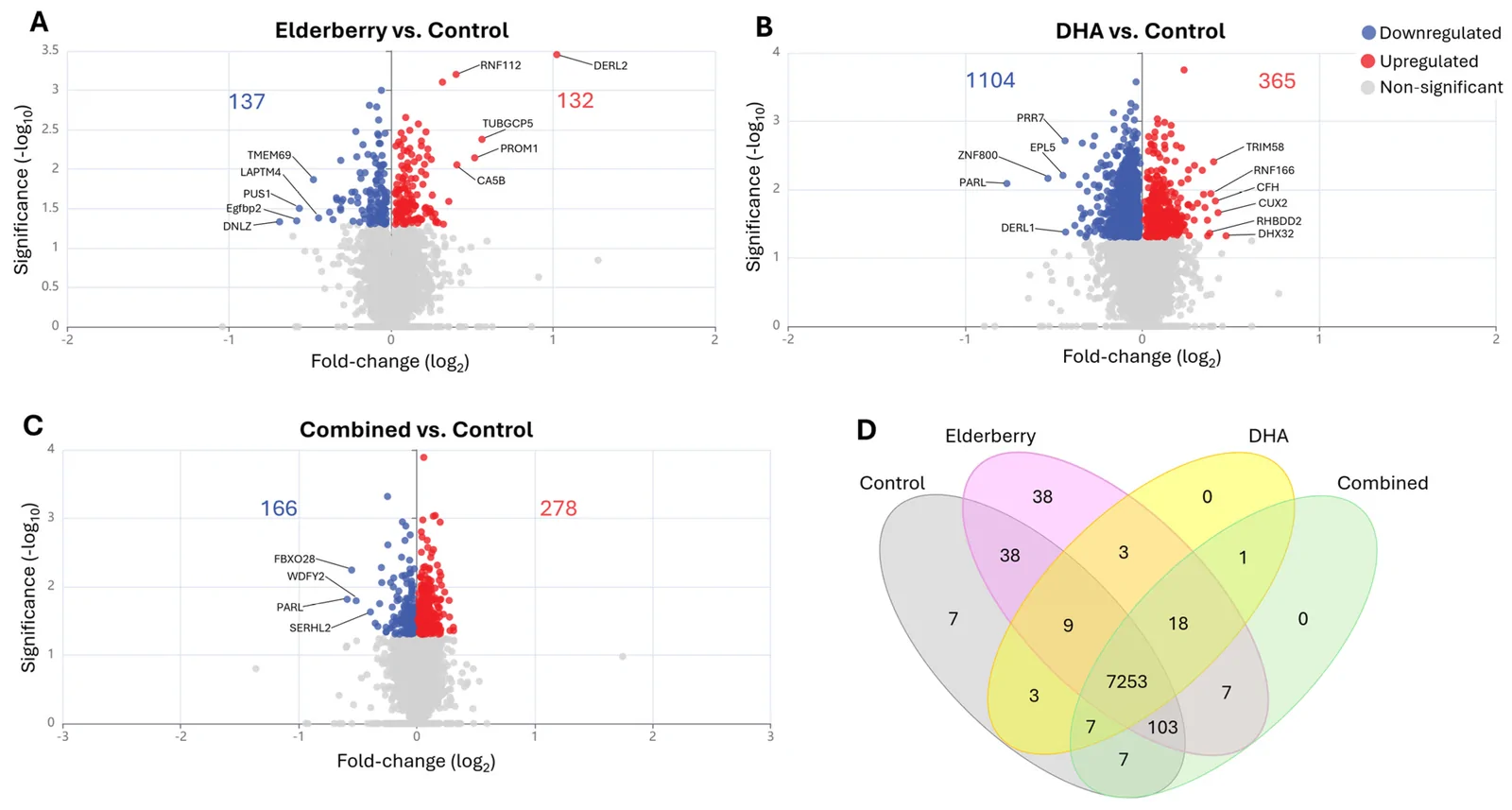
Global proteomic profiling and identification of differentially expressed proteins. (**A**–**C**) Volcano plots illustrating DEPs in the global proteome for (**A**) Elderberry vs. Control, (**B**) DHA vs. Control, and (**C**) Combined vs. Control. The *x*-axis represents log_2_ fold change (FC) (expression log ratio), and the *y*-axis represents the statistical significance (−log10 *p*-value). Red dots indicate proteins with increased abundance, whereas blue dots indicate proteins with decreased abundance based on the exploratory criterion of *p* ≤ 0.05. Proteins meeting the combined thresholds of *p* ≤ 0.05 and |log_2_ (FC)| ≥ 0.38 are highlighted and labeled. (**D**) Venn diagram showing the overlap of the identified proteins among the four groups.

Global proteomic analysis identified 7494 proteins among all experimental groups. Differential expression analysis visualized by volcano plots (Figure 1A–C) revealed dietary-specific patterns of proteomic regulation, with each dietary supplementation group showing a distinct distribution and number of significantly altered proteins. Compared with controls, the elderberry group exhibited moderate changes, with 132 upregulated and 137 downregulated proteins (Figure 1A). In contrast, DHA supplementation produced the most extensive proteomic remodeling, characterized by 365 upregulated and 1104 downregulated proteins (Figure 1B). The combined group displayed a distinct response, with 278 upregulated and 166 downregulated proteins (Figure 1C).

Among proteins meeting *p* ≤ 0.05, those with |log_2_ (FC)| ≥ 0.38 were labeled in Figure 1 to highlight representative proteins with larger abundance changes, including DERL2 and RNF112 in the elderberry group, PRR7 and TRIM58 in the DHA group, and FBXO28 and WDFY2 in the combined group. The Venn diagram (Figure 1D) showed that 7253 proteins were shared by all four groups, indicating substantial overlap in the detected proteome. A limited number of proteins were uniquely detected, including thirty-eight in the elderberry group and seven in the control, whereas no unique proteins were detected in the DHA or combined groups.

GO functional-enrichment analysis of the global proteomic dataset identified distinct top-ranked Biological Process, Cellular Component, and Molecular Function terms among the elderberry, DHA, and combined groups relative to control. The five GO terms with the lowest raw *p* values within each category and dietary comparison, together with their FDR-adjusted q values, are presented in Supplementary Table S1.

### 3.3. Phosphoproteomic Analysis Reveals Convergent Neuroplasticity Pathways

**Figure 2:**
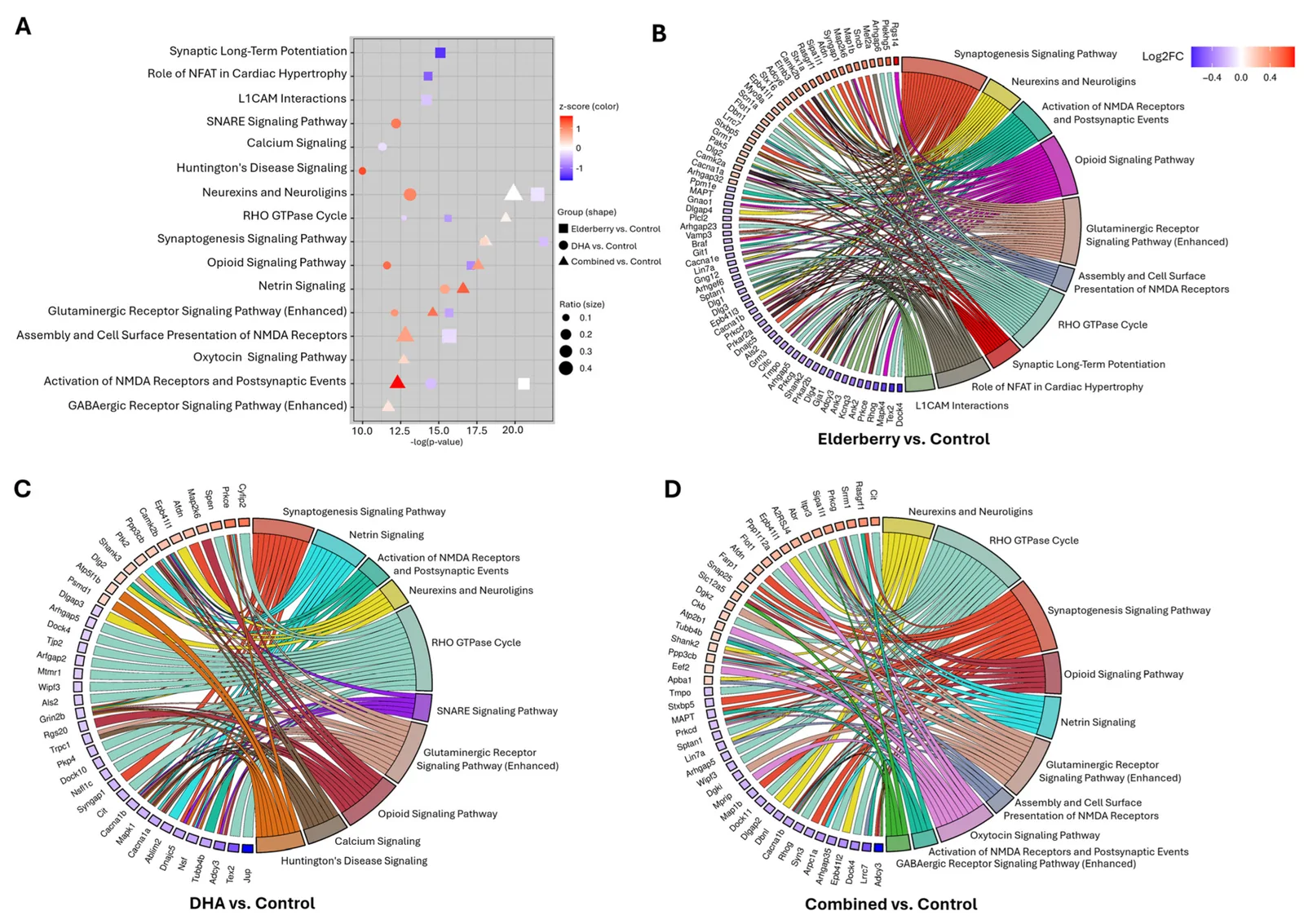
Phosphoproteomic analysis of pathway enrichment. (**A**) Bubble plot of enriched canonical pathways based on phosphoproteomic data. The *x*-axis represents pathway-enrichment significance (−log10 *p*-value), and the color scale indicates the IPA-predicted activation z-score (red: activation; blue: inhibition). Symbol shape denotes the comparison group: squares represent elderberry (EB) vs. control, circles represent DHA vs. control, and triangles represent combined EB + DHA vs. control. Bubble size corresponds to the proportion of identified proteins mapped to each pathway (ratio). (**B**–**D**) Chord diagrams illustrating the relationship between differentially expressed phosphoproteins and enriched canonical pathways for (**B**) Elderberry vs. Control, (**C**) DHA vs. Control, and (**D**) Combined vs. Control. In each diagram, the right semicircle represents the canonical pathways, and the left semicircle represents individual proteins, colored according to log_2_-fold change (Log_2_ (FC); red: upregulated; blue: downregulated). Ribbons connect proteins to their associated pathways. Pathway enrichment was defined using a significance threshold of *p* ≤ 0.05. Chord diagram visualization includes proteins meeting an additional fold-change cutoff (|log_2_ (FC)| ≥ 0.2).

Phosphoproteomic profiling followed by IPA canonical pathway enrichment analysis revealed dietary-specific phospho-signaling signatures, while also identifying substantial convergence among neuroplasticity-related pathways (Figure 2A). The bubble plot showed that enriched pathways were primarily associated with synaptic function, receptor signaling, cytoskeletal regulation, and neuronal communication. Elderberry supplementation exhibited a predominantly predicted inhibitory or neutral profile, with nine of the top ten enriched pathways showing negative z-scores, including synaptic long-term potentiation, role of NFAT in cardiac hypertrophy, the RHO GTPase cycle, glutamatergic receptor signaling, and NMDA receptor-related pathways. In contrast, DHA supplementation and the combined group displayed a more predicted activation-oriented profile, particularly for pathways related to synaptogenesis signaling, neurexin/neuroligin signaling, netrin signaling, opioid signaling, glutamatergic receptor signaling, and NMDA receptor activation and post-synaptic events. Despite these differences, several core neuroplasticity-related pathways were consistently enriched in all supplementation groups, including synaptogenesis signaling, glutamatergic receptor signaling, and NMDA receptor activation, as well as additional pathways such as neurexin/neuroligin signaling, the RHO GTPase cycle, and opioid signaling (Figure 2B–D). These observations indicate that all dietary interventions were associated with changes in phosphorylation networks associated with synaptic remodeling and neuronal signaling.

Chord diagram analysis further elucidated the phosphoprotein–pathway relationships underlying these enrichment patterns (Figure 2B–D). Using a pathway enrichment threshold of *p* ≤ 0.05 and a chord-diagram display cutoff of |log_2_ (FC)| ≥ 0.2, the diagrams revealed substantial overlap in pathway-associated phosphoproteins among dietary groups while highlighting distinct pathway signatures for each intervention. Synaptogenesis signaling, neurexin/neuroligin signaling, glutamatergic receptor signaling, NMDA receptor activation, opioid signaling, and RHO GTPase signaling were represented in multiple supplementation groups. Beyond these shared pathways, the elderberry group showed prominent associations with synaptic long-term potentiation, NFAT signaling, and L1CAM interactions (Figure 2B). The DHA group showed additional connectivity with SNARE signaling, calcium signaling, and Huntington’s disease signaling (Figure 2C). The combined group exhibited further representation of netrin signaling, NMDA receptor assembly pathways, oxytocin signaling, and GABAergic receptor signaling (Figure 2D). In all three diagrams, recurrent phosphoproteins, including MAPT, DLG4, and glutamate receptor-associated protein GRIK2, appeared in multiple synaptic and receptor-mediated pathways. These observations suggest that elderberry, DHA, and their combined supplementation were associated with partially overlapping phosphorylation patterns involving synaptic organization, receptor trafficking, and activity-dependent neuroplasticity.

### 3.4. Predicted Upstream Kinase Signatures and Their Relationships with Behavioral Outcomes

**Figure 3:**
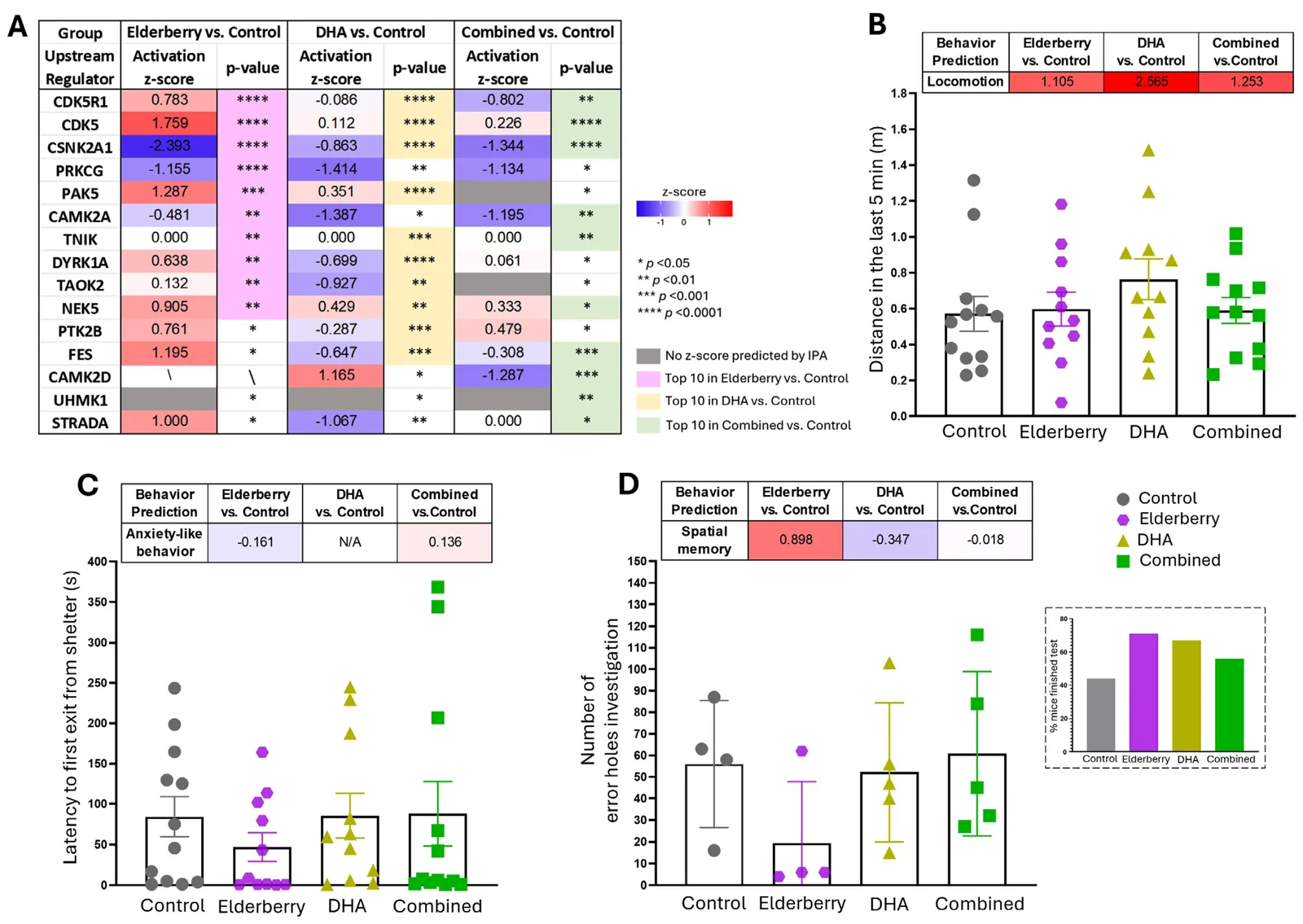
IPA-based predicted upstream-kinase analysis and behavioral-function annotations compared with measured behavioral outcomes across dietary interventions. (**A**) Upstream-regulator analysis showing predicted key kinase-associated signatures across dietary groups. The heatmap displays IPA activation z-scores and corresponding enrichment significance values for Elderberry, DHA, and Combined (EB + DHA) groups relative to Control group. The color scale indicates predicted activation states (red: activation, z > 0; blue: inhibition, z < 0). Shaded cells highlight top-ranked upstream regulators within each comparison group. (**B**–**D**) IPA-predicted behavioral function activation z-scores are shown above independently measured behavioral outcomes. These comparisons are descriptive and do not represent experimental validation of the IPA predictions. Unmarked group differences did not reach statistical significance. (**B**) Locomotor activity. The upper panel shows IPA-predicted activation z-scores for the behavioral function “Locomotion” across groups. The lower bar-and-scatter plot shows total distance traveled during the final 5 min of the OFT. (**C**) Anxiety-like behavior. The upper panel shows IPA-predicted activation z-scores for the behavioral function corresponding to anxiety-related activity. The lower bar-and-scatter plot shows latency to first exit from the shelter in the emergence test. (**D**) Spatial learning and memory. The upper panel shows IPA-predicted z-scores for learning- and memory-related functional annotations. The inset bar chart represents the percentage of mice completing the Barnes maze within 5 min. The lower bar-and-scatter plot shows the number of error-hole investigations on Day 2, Trial 1, reflecting spatial learning performance. Data are presented as mean ± SEM. Measured behavioral outcomes were analyzed by one-way ANOVA followed by Dunnett’s multiple comparisons test. IPA-predicted activation z-scores were compared descriptively with the measured outcomes and were not included in the statistical analysis. Significant differences are indicated as follows: * *p* < 0.05, ** *p* < 0.01, *** *p* < 0.001, **** *p* < 0.0001 vs. control.

Upstream regulator analysis identified distinct kinase-associated regulatory signatures among the dietary groups, suggesting partially overlapping but dietary-specific upstream networks (Figure 3A). The heatmap revealed both predicted activation and inhibition of upstream regulators, with several regulators showing group-dependent directionality based on IPA activation z-scores. Several regulators, including CSNK2A1 (Casein Kinase 2 Alpha 1) and PRKCG, exhibited predicted inhibition in all supplementation groups, suggesting a shared molecular response to dietary interventions. In contrast, the elderberry group displayed a distinct regulatory signature characterized by prediction in activation of CDK5 (z = 1.76) and its neuronal activator CDK5R1 (z = 0.78), a pattern that differs from that observed in the DHA group. DHA supplementation was instead associated with CAMK-related signaling pathways, including CAMK2D and STRADA, which showed negative IPA activation z-scores in other groups. Notably, CAMK2A had negative activation z-scores in all groups. These findings indicate divergent-predicted kinase-associated signatures, particularly involving CDK5- and CAMK-related signaling pathways. The combined group showed a distinct profile, including both predicted inhibitory and activating regulatory signals, suggesting that its phospho-signaling profile did not simply mirror that of either individual intervention.

To determine whether the predicted upstream-phosphoproteomic signatures showed directional relationships with behavior outcomes, IPA-annotation predictions were compared with corresponding measures of locomotor activity, anxiety-like behavior, and spatial learning and memory (Figure 3B–D). For locomotor activity (Figure 3B), IPA predicted positive activation z-scores for all supplementation groups relative to the control group, with the strongest predicted activation in the DHA group (z = 2.57). The DHA group also showed a numerically higher distance traveled during the final 5 min of the open field test, although the group difference did not reach statistical significance. No significant group differences were observed during the initial 5 min (Figure S2A). Center zone activity, measured as the number of line crossings, did not differ among groups (Figure S2B).

For anxiety-like behavior (Figure 3C), a slight decrease was predicted in the elderberry group (z = −0.16), whereas a mild increase was predicted in the combined group (z = 0.14). These predictions showed partial directional correspondence with numerical differences in the emergence test, including reduced lower latency-related anxiety-like behavior in the elderberry group and a numerical increase in the combined group; neither difference reached statistical significance.

For spatial learning and memory (Figure 3D), improved performance was predicted in the elderberry group (z = 0.90), whereas the DHA (z = −0.30) and combined (z = −0.02) groups showed neutral or negative trends. Behaviorally, elderberry and DHA groups showed numerical differences in Barnes maze performance, including a higher percentage of mice completing the task within 5 min and fewer error-hole investigations on Day 2, Trial 1, whereas the combined group showed an intermediate and variable response. Together, these findings demonstrate that the relationships between IPA-predicted functions and measured-behavioral outcomes varied across dietary groups and behavioral domains involving locomotion, anxiety-like behavior, and spatial learning.

### 3.5. Elderberry and DHA Supplementation Reduce Anxiety-like Behavior in the Elevated Plus Maze (EPM) Test

**Figure 4:**
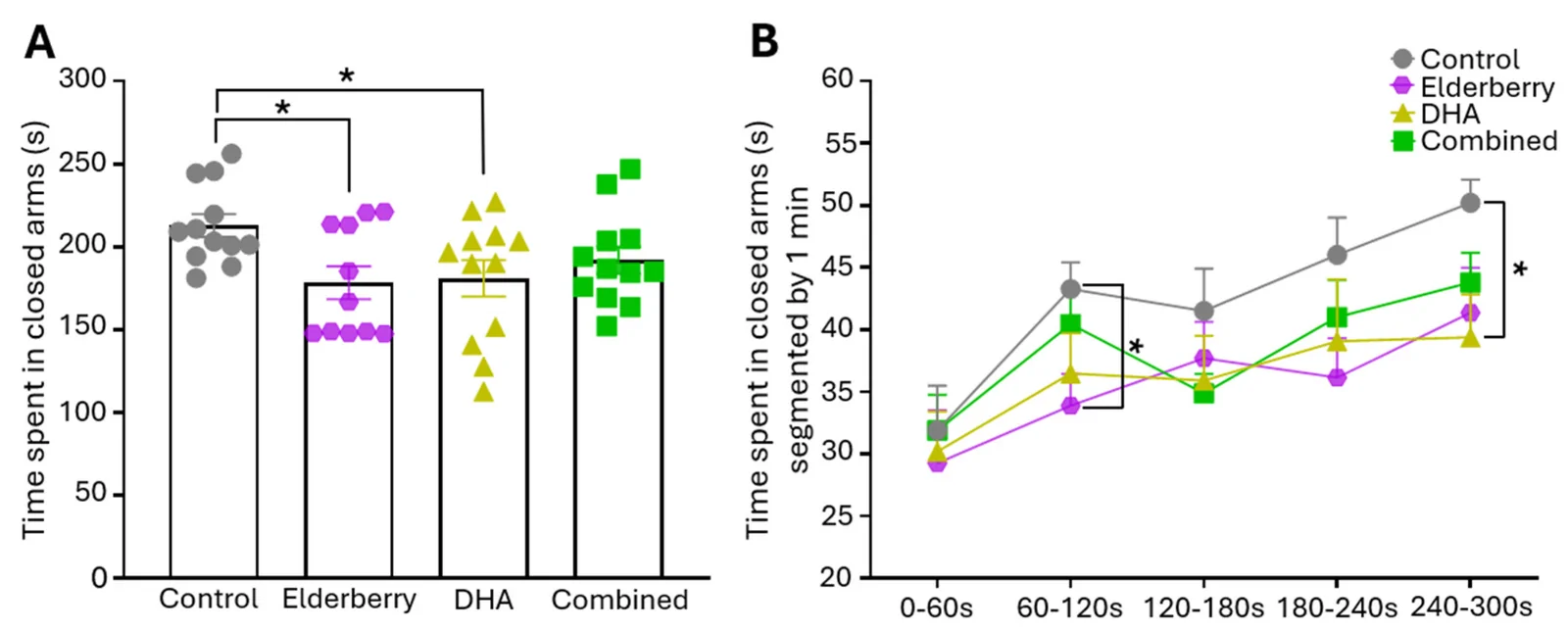
Effects of dietary supplementation on anxiety-like behavior assessed by the elevated plus maze test (EPM). (**A**) Total time spent in closed arms during the EPM test across groups. Both Elderberry and DHA supplementation significantly reduced closed-arm time compared with Control, whereas the combined group showed a non-significant decreasing trend. (**B**) Temporal analysis of closed-arm occupancy, showing time spent (s) in closed arms segmented into 1-min intervals over the 5-min trial. A significant reduction was observed in the EB group during the 60–120 s interval, while DHA and combined groups showed non-significant decreases. Data are presented as mean ± SEM. Panel A was analyzed by one-way ANOVA, and Panel B by two-way ANOVA, both followed by Dunnett’s multiple-comparisons test. * *p* < 0.05 versus Control.

In the EPM test, both elderberry and DHA supplementation significantly reduced anxiety-like behavior compared with the control diet group. Mice in these groups spent less time in closed arms (*p* < 0.05; Figure 4A). The combined group also showed a decreasing trend, although this did not reach statistical significance. Temporal analysis of the trial, segmented into 1-min intervals, revealed a significant reduction in closed-arm occupancy during the 60–120 s interval compared with the control-diet group (*p* < 0.05, Figure 4B). The DHA and combined groups also exhibited lower occupancy during this period, although these changes did not reach statistical significance.

### 3.6. Elderberry-Associated Changes in Spatial Learning

**Figure 5:**
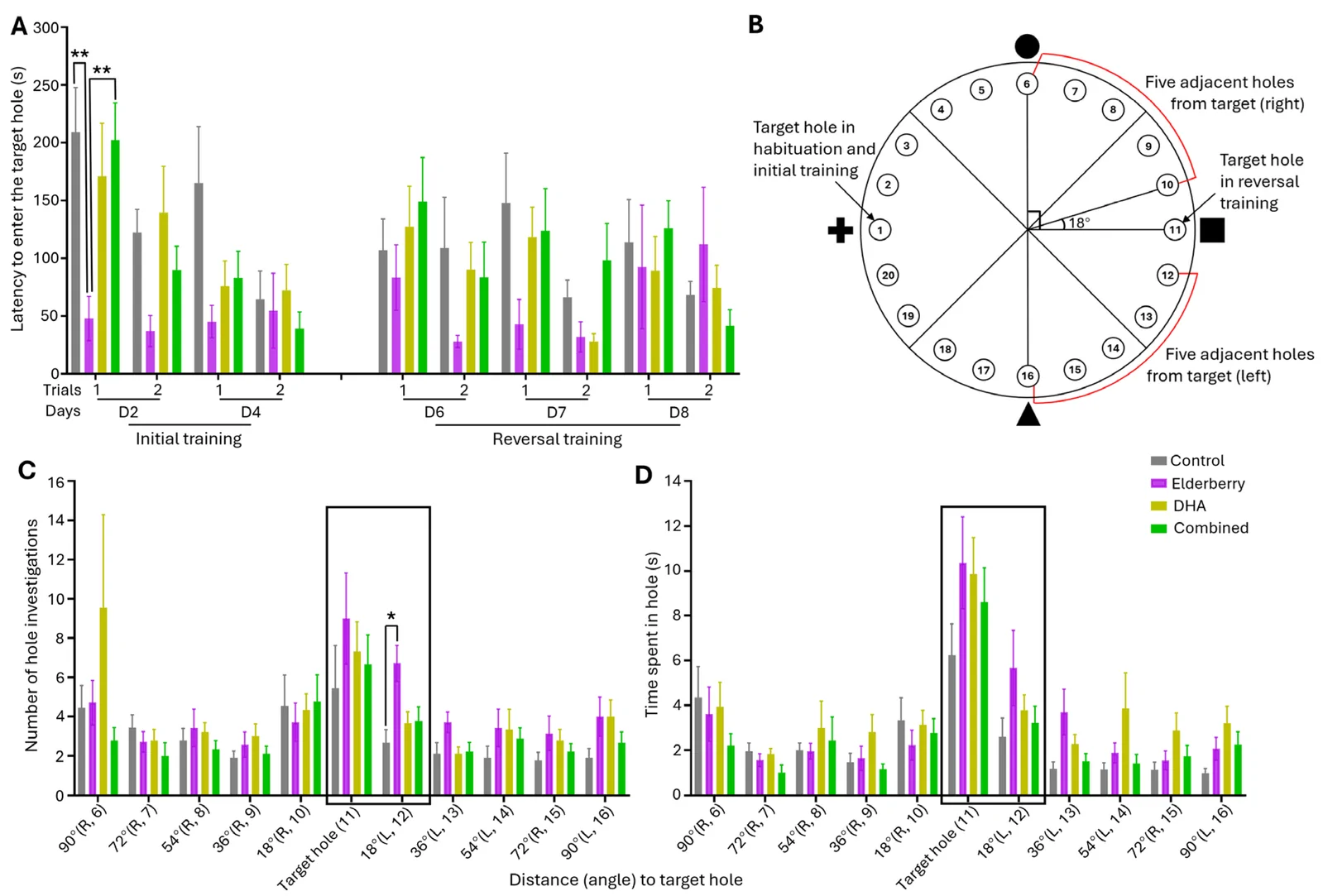
Effects of dietary supplements on spatial learning and memory assessed using Barnes maze. (**A**) Latency to enter the target hole during initial training (Days 2 and 4) and reversal training (Days 6–8). (**B**) Barnes maze layout indicating the reversal target and adjacent zones. Four visual cues (**+**, ⯀, ⯅, •) were placed on surrounding curtains to facilitate navigation. (**C**,**D**) Barnes maze probe test on Day 9, 24 h after the final reversal trial, assessed by the number of hole investigations (**C**) and time spent at each hole (**D**). Time spent at each hole location. Data are presented as Mean ± SEM. Panels (**A**,**C**,**D**) were analyzed using two-way repeated-measures ANOVA followed by Tukey’s multiple-comparisons test. * *p* < 0.05 and ** *p* < 0.01. Initial training: control, *n* = 6; elderberry, *n* = 4; DHA, *n* = 6; combined, *n* = 8. Reversal training: control, *n* = 8; elderberry, *n* = 5; DHA, *n* = 9; combined, *n* = 9. The probe test: control, *n* = 9; elderberry, *n* = 7; DHA, *n* = 9; combined, *n* = 9.

Spatial learning and memory were assessed using the Barnes maze (Figure 5 and Figure S3). During initial acquisition, mice―one in control, two in elderberry, one in DHA, and one in combined-group―were classified as phase non-completers. Among mice included in the acquisition-latency analysis, the elderberry group showed a significantly shorter latency to locate the escape hole on Day 2, Trial 1 than the control and combined group (** *p* < 0.01). These differences were not consistently observed across subsequent acquisition trials (Figure S3). During reversal training (Days 6–8), one control and two elderberry mice were classified as phase non-completers. Among mice included in the reversal-latency analysis, no significant differences were observed among dietary groups (Figure 5A). In the probe test (Day 9), elderberry-fed mice demonstrated a greater preference for the target area, reflected by increased hole investigations at the target and adjacent positions, with a significant difference at one position adjacent to the target (*p* < 0.05, Figure 5C), indicating increased target-directed search during the probe test. The elderberry group also spent numerically more time near the target location, suggesting a more precise spatial memory trace (Figure 5D). Together, these findings indicate a shorter initial-acquisition latency among the elderberry-fed mice included in the latency analysis and increased target-directed search during the probe test, alongside variability in phase completion and no significant group differences during reversal training.

### 3.7. Striatal Fatty Acid Composition in the Brain

**Figure 6:**
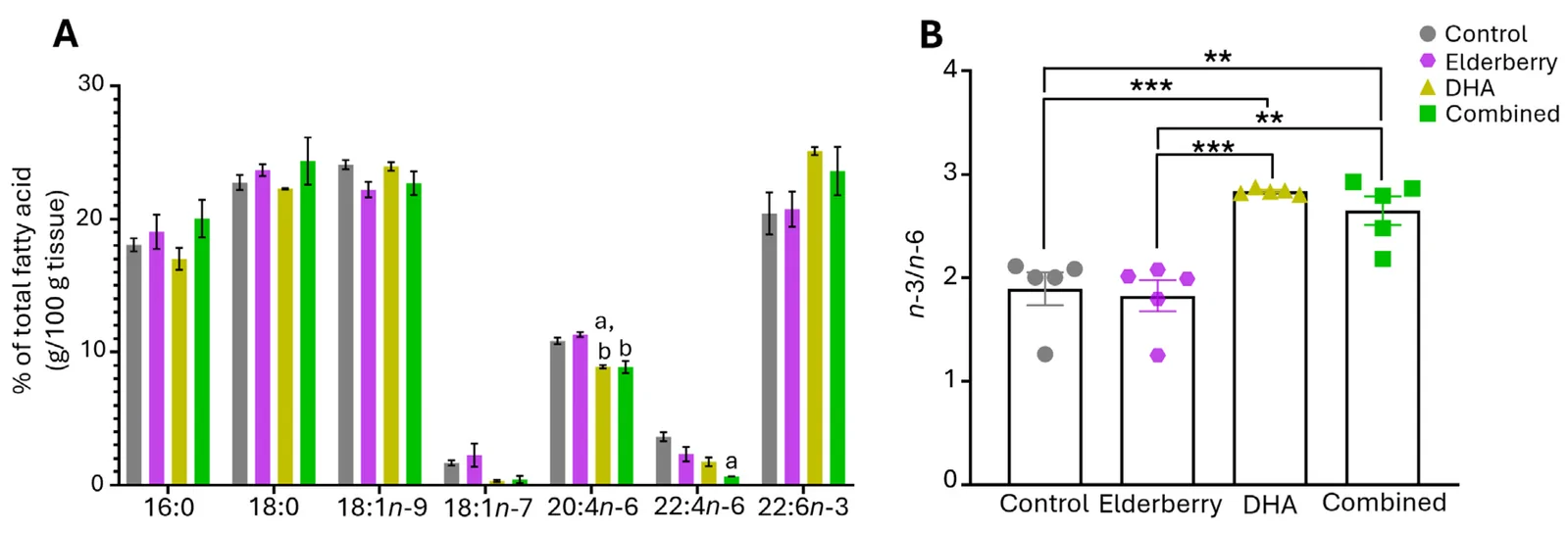
Effects of dietary supplementation on striatal fatty-acid composition. (**A**) Relative abundance of major fatty acids in the left striatum, expressed as a percentage of total fatty acids (g/100 g tissue), including 16:0, 18:0, 18:1*n*-9, 18:1*n*-7, 20:4*n*-6, 22:4*n*-6, and 22:6*n*-3, across control, elderberry, DHA, and combined groups. DHA supplementation increased DHA (22:6*n*-3) levels and reduced 20:4*n*-6, 22:4*n*-6, and 18:1*n*-7. Letters indicate significant pairwise differences determined by Tukey’s multiple-comparisons test: “a,” compared with the control group; “b,” compared with the elderberry group (*p* < 0.05). (**B**) Ratio of omega-3 to omega-6 fatty acids (*n*-3/*n*-6) across groups. Data are presented as mean ± SEM. Statistical analyses were performed using one-way ANOVA followed by Tukey’s multiple-comparisons test. Asterisks indicate significant pairwise differences (** *p* < 0.01, *** *p* < 0.001).

Fatty acid composition was measured in the left striatum as cortical tissue was allocated to proteomic and phosphoproteomic analyses (Figure 6A). The major fatty acids identified included 16:0, 18:0, 18:1*n*-9, 20:4*n*-6, and 22:6*n*-3 (Figure 6A). DHA supplementation resulted in reduced levels of *n*-6 fatty acids (e.g., 20:4*n*-6 and 22:4*n*-6) and increased levels of *n*-3 fatty acids, mainly 22:6*n*-3 (Figure 6A). There was also a decrease in levels of 18:1*n*-7 in the DHA and combined groups, albeit the data did not reach statistical significance. In contrast, elderberry supplementation produced only limited changes in fatty acid composition as compared with the control group (Figure 6A).

To evaluate the balance between omega-3 and omega-6 fatty acids, the *n*-3/*n*-6 ratio was calculated for each group (Figure 6B). One-way ANOVA revealed a significant effect of diet on the *n*-3/*n*-6 ratio (F(3,16) = 15.69, *p* < 0.0001). Post hoc Tukey’s multiple-comparisons test showed that the *n*-3/*n*-6 ratio was significantly increased in both the DHA and combined groups compared with the control and elderberry groups (Figure 6B). No significant difference was observed between control and elderberry groups or between the DHA and combined groups. These findings indicate that DHA supplementation produced the most prominent alterations in striatal fatty-acid composition, whereas elderberry alone did not significantly alter the measured fatty-acid profile or further increase the DHA-associated elevation in the *n*-3/*n*-6 ratio.

## 4. Discussion

This study combined global proteomics profiling, IPA analysis of phosphoproteomic data, functional analysis, fatty-acid composition, and behavioral assessments to evaluate the effects of elderberry and DHA supplementation and examine the relationships between molecular signatures and neurobehavioral outcomes in aging mice. Global-proteomic profiling characterized dietary-specific changes in protein abundance, whereas the phosphoproteomic dataset was analyzed using IPA to identify enriched pathways, predict upstream regulators, and infer behavior-related functions. These analyses particularly implicated signaling networks involved in synaptic organization, glutamatergic signaling, NMDA-receptor-associated postsynaptic processes, and kinase-regulated phosphorylation.

IPA-predicted behavioral functions were compared descriptively with the observed outcomes, including locomotor activity, anxiety-like behavior, and spatial learning, with varying degrees of directional correspondence across outcomes. This comparison provides functional context for interpreting the identified molecular signatures.

At the protein-abundance level, the dietary groups exhibited distinct proteomic responses (Figure 1A–C). Elderberry supplementation produced a more targeted profile, highlighted by differential expression of proteins associated with protein quality control and proteostasis. The increased abundance of DERL2 and RNF112 suggests modulation of proteostasis in the aged cortex. DERL2 is a component of the endoplasmic-reticulum-associated degradation (ERAD) pathway [61,62,63], and its upregulation may reflect enhanced clearance of misfolded or damaged proteins, thereby reducing ER stress and limit maladaptive unfolded protein response pathways that contribute to neuronal vulnerability during aging [64]. In contrast, DHA supplementation produced broad proteomic remodeling, with more than 1400 differentially expressed proteins, suggesting widespread effects on protein networks, particularly those related to membrane-associated and synaptic processes (Figure 1B). In the combined group, the presence of FBXO28 further suggests involvement of the ubiquitin-proteasome system, indicating a protein-abundance pattern that differed from those observed with either intervention alone and may involve protein quality-control processes relevant to age-associated proteotoxic stress (Figure 1C) [65].

Phosphoproteomic profiling shifted the focus from protein abundance to functional regulation of synaptic signaling pathways. Notably, pathway enrichment analysis revealed divergent activation patterns, with elderberry-associated networks showing predominantly negative activation z-scores, whereas DHA-associated pathways tended to show positive activation z-scores. CAMK2A and CAMK2B emerged as central hubs, consistent with the critical role of CaMKII-dependent phosphorylation in synaptic plasticity and long-term potentiation [66,67,68]. In addition, reduced phosphorylation of MAPT/Tau was observed in the elderberry and combined groups. Because aberrant Tau phosphorylation is associated with microtubule instability, impaired axonal transport, and synaptic dysfunction during aging and neurodegeneration [69,70], reduced MAPT/Tau phosphorylation may be associated with altered cytoskeletal and synaptic regulation, although this interpretation requires further validation. This interpretation consists of concurrent changes in synaptic scaffold and receptor-related proteins, including GRIK2 and DLG4, which are involved in glutamatergic signaling and synaptic structure. The overlap of key phosphoproteins across pathways further suggests that these interventions were associated with interconnected signaling networks rather than isolated pathways.

Comparative pathway analysis identified both shared and intervention-associated molecular patterns. Synaptogenesis, glutamatergic receptor signaling, NMDA receptor-associated processes, neurexin/neuroligin signaling, and RHO GTPase signaling were represented across the supplementation groups (Figure 2B–D). Elderberry showed additional associations with long-term potentiation, NFAT signaling, and L1CAM interactions, whereas DHA showed additional associations with SNARE and calcium signaling. The combined group retained several shared synaptic pathways while showing further representation of netrin signaling, NMDA receptor assembly, oxytocin signaling, and GABAergic receptor signaling. Thus, the combined intervention was associated with a partially overlapping but nonidentical molecular signature rather than simply reproducing individual intervention.

Upstream regulator analysis identified candidate regulatory signatures associated with these phosphoproteomic changes (Figure 3A). CDK5–CDK5R1 signaling was associated with groups receiving elderberry supplementation, whereas DHA supplementation was more closely linked to CAMK-related pathways, including CAMK2D and STRADA. Although CDK5 is often associated with pathological Tau phosphorylation, it also plays essential roles in neuronal function and synaptic plasticity [71]. These findings suggest that elderberry and DHA were associated with partially distinct molecular signatures, with elderberries linked to cytoskeletal and Tau-related regulatory pathways and DHA linked to CaMK-associated synaptic signaling and lipid metabolism. The predicted inhibition of CSNK2A1 across all supplementation groups may represent a shared candidate regulatory response associated with reduced stress- or inflammatory-related signaling [72,73].

The observed molecular and pathway-level changes showed varying degrees of directional correspondence with behavioral outcomes, including locomotor activity, anxiety-like behavior, and spatial learning. This comparison provides functional context for interpreting molecular signatures. Predicted increases in locomotor activity, particularly in the DHA group, were directionally reflected by a numerical increase in distance traveled during the later phase of open field testing (last 5 min of the 10-min trial; Figure 3B), after the initial novelty-driven exploratory phase. Similarly, predicted anxiety-related functions showed partial directional correspondence with anxiety-like behavior measured in the emergence test and elevated plus maze (Figure 3C and Figure 4). However, without further orthogonal validation these associations may not establish that elderberry directly altered the underlying neural circuits. The relationship between the predicted functions and behavioral performance was examined in the Barnes maze (Figure 3D, Figure 5 and Figure S3). Barnes maze performance varied within the dietary groups. Among mice included in the latency analysis, elderberry supplementation was associated with a shorter latency during early acquisition, but this difference was not consistently maintained across subsequent acquisition trials, and no significant differences were detected during reversal training. The phase-completion results also indicate that the behavioral response was not uniform across elderberry-fed mice. In the probe test, elderberry-fed mice showed increased target-directed search, suggesting increased target-directed search. Previous works have shown similar association between anthocyanin supplementation with improved spatial performance and changes in synaptic-plasticity-related proteins in aged animals and neurological-disease models [22,74]. The combined group showed an intermediate and variable behavioral response despite its distinct phosphoproteomic profile. This divergence may reflect differences between cortical molecular signatures and behavioral outcomes involving distributed neural circuits. Thus, the combined-group findings indicate a nonidentical molecular response but did not provide evidence of an additive or synergistic benefit in spatial learning.

Differences between the IPA-predicted functions and measured behavioral outcomes may reflect the computational nature of the predictions, biological variability, differences in sample size, the regional specificity of the cortical phosphoproteomic analysis, and the involvement of distributed neural circuits in behavioral performance.

These behavioral observations showed exploratory directional correspondence with the elderberry-associated synaptic and cytoskeletal signatures, including predicted CDK5–MAPT-related regulation. Together, these findings indicate directional relationships between the observed molecular signatures and behavioral outcomes.

Fatty acid profiling provided an additional biochemical context for interpreting these findings. DHA supplementation altered striatal fatty-acid composition, characterized by increased DHA (22:6*n*-3), reduced levels of *n*-6 fatty acids, including arachidonic acid (ARA, 20:4*n*-6) and adrenic acid (22:4*n*-6), and significantly elevated *n*-3/*n*-6 ratio. These findings support previous studies demonstrating that dietary DHA could modulate (*n*-3)/(*n*-6) fatty-acid balance in the brain [75]. Because ARA serves as a precursor of pro-inflammatory lipid mediators, its reduction may contribute to a more favorable lipid environment for limiting inflammatory signaling in the aging brain [76]. The reciprocal balance between DHA and ARA has been associated with distinct roles in lipid-mediated signaling and inflammatory regulation in the central nervous system [77]. In contrast, elderberry supplementation alone produced only minor changes in striatal fatty-acid composition and did not significantly alter the *n*-3/*n*-6 ratio, suggesting a molecular profile more prominently associated with protein signaling networks than with lipid remodeling. Fatty acids are incorporated into phospholipids, which are major structural components of cell membranes; therefore, further studies examining how changes in the *n*-3/*n*-6 fatty-acid balance affect membrane phospholipid composition may provide additional mechanistic insight. Together, these findings suggest that elderberry and DHA were associated with partially overlapping but distinct molecular responses.

### 4.1. Limitations of the Work

This study involved only male C57BL/6J mice, thus there was a limited ability to compare sex differences. In addition, the study used normal middle-aged mice rather than a model of Alzheimer’s disease or pathological neurodegeneration. The proteomic and phosphoproteomic analyses included five biological replicates per group, which was appropriate for discovery-oriented mass-spectrometry profiling. Nevertheless, biological variability and limited statistical power should be considered when interpreting smaller-magnitude changes. The differential-abundance analyses on proteomic- and phosphoproteomic data used an exploratory *p* ≤ 0.05 criterion without multiple-testing adjustment, and the findings were not independently validated using targeted protein, phosphosite, kinase-activity assays, or histological assays. Therefore, individual molecular associations should be interpreted as candidate findings. The IPA-derived pathway and upstream-regulator outputs represent computational predictions. Accordingly, the MAPT/Tau-, CDK5-, and CAMK2-associated findings should be interpreted as candidate molecular signatures rather than confirmed mechanisms.

### 4.2. Future Research Directions

Future studies should include appropriately powered cohorts of age-matched female mice to evaluate sex-dependence molecular and behavioral responses, including potential influences of hormonal status. Factorial studies are also needed to formally evaluate elderberry × DHA interactions. Selected proteins and phosphorylation sites should be validated using targeted quantitative assays, such as phospho-specific immunoblotting, targeted mass spectrometry, or immunohistochemistry, in an independent cohort. Studies in relevant models of pathological neurodegeneration will be required before extending these findings to Alzheimer’s disease or related disorders.

## 5. Conclusions

In summary, this study demonstrates that elderberry, DHA, or their combination are associated with distinct molecular and differing behavioral-response patterns in aging mice. Mice receiving elderberry supplementation showed reduced anxiety-like behavior, a shorter latency during early the initial training phase among mice included in the latency analysis, and increased target-directed search during the Barnes maze probe test, although Barnes maze performance varied within the group. DHA supplementation showed a numerical increase in locomotor activity during the final 5 min of the open field test, although the difference did not reach statistical significance. No significant group differences in total distance traveled were observed during the initial 5-min exploratory phase. Global proteomic profiling revealed dietary-specific changes in protein abundance, while IPA analysis of the phosphoproteomic dataset identified interconnected signaling networks involving synaptic plasticity, glutamatergic transmission, and kinase-regulated phosphorylation.

At the molecular level, elderberry supplementation was associated with a predicted CDK5-CDK5R1 regulatory signature and reduced MAPT/Tau phosphorylation at selected sites, suggesting candidate associations with cytoskeletal and synaptic regulation. In contrast, DHA supplementation was associated with a predicted CAMK-related signature and changes in fatty-acid composition, suggesting a distinct molecular profile involving synaptic signaling and lipid composition. Predicted inhibition of CSNK2A1 in all supplementation groups may represent a shared candidate regulatory response.

Notably, combined elderberry + DHA supplementation was associated with partially overlapping but nonidentical molecular signatures compared with the individual interventions. The combined-group profile included Tau-related cytoskeletal pathways, CAMK-associated synaptic signaling, and lipid remodeling; however, these descriptive findings do not establish an interaction between the two supplements.

Together, the results support further investigation of elderberry and DHA as dietary strategies targeting molecular pathways and neurobehavioral outcomes associated with brain aging.

## Data Availability

All data supporting the findings of this study are available within the article and its Supplementary Material. The raw datasets and additional materials are available from the corresponding author upon reasonable request.

## Supplementary Materials

The following supporting information can be downloaded at https://www.mdpi.com/article/10.3390/nu18172802/s1, Figure S1: Body weight and food intake during the 12-week dietary intervention; Figure S2: Locomotor and center-zone activity during the initial phase of the open field test (OFT); Figure S3: Barnes maze latency during habituation and initial acquisition training; Table S1: Top-ranked GO functional-enrichment terms for proteins meeting the exploratory differential-abundance criterion in the elderberry, DHA, and combined groups relative to control.
