# Supplementary material for: Proteomic and Phosphoproteomic Signatures Link Molecular Remodeling to Behavioral Outcomes Following Elderberry and DHA Supplementation in Aging Mice
Source: Nutrients. 2026 Aug 27;18(17):2802. doi: 10.3390/nu18172802 (PMC13567599; doi:10.3390/nu18172802)
Supplement: Supplementary file 1 [file nutrients-18-02802-s001.zip › nutrients-4481032-supplementary.pdf]

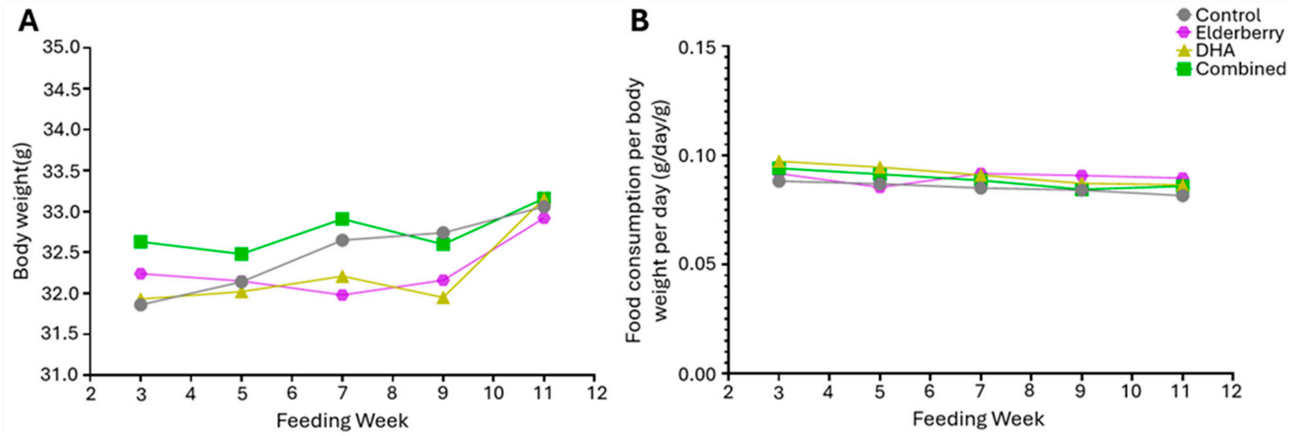


**Figure S1.** Body weight and food intake during the 12-week dietary intervention (A) Body weight (g) measured throughout the feeding period. (B) Food intake normalized to body weight (g/day/g body weight). Data are presented as mean ± SEM; No significant differences were detected among groups by two-way ANOVA.

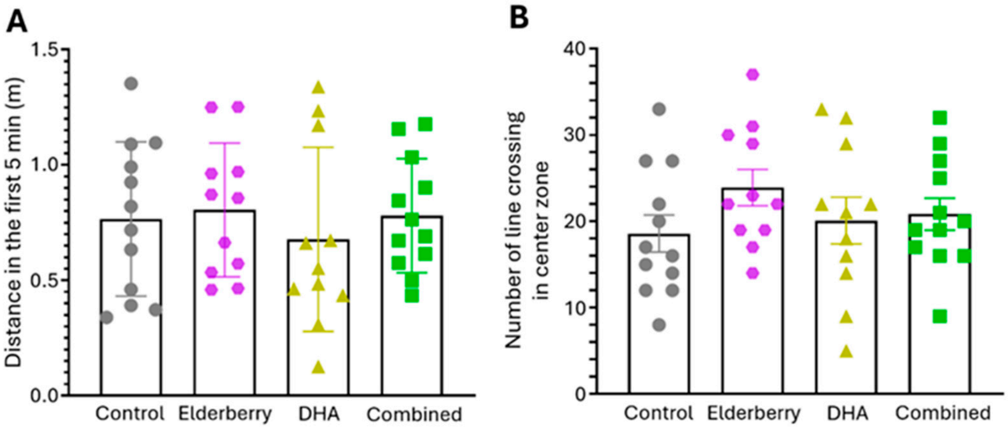


**Figure S2.** Locomotor and center-zone activity during the initial phase of the open field test (OFT). (A) Total distance traveled. (B) Center-zone activity, quantified as the number of line crossings, during the first 5 minutes of the OFT. Data are presented as mean ± SEM and were analyzed by one-way ANOVA followed by Tukey's multiple comparisons test. Individual data points represent individual animals.

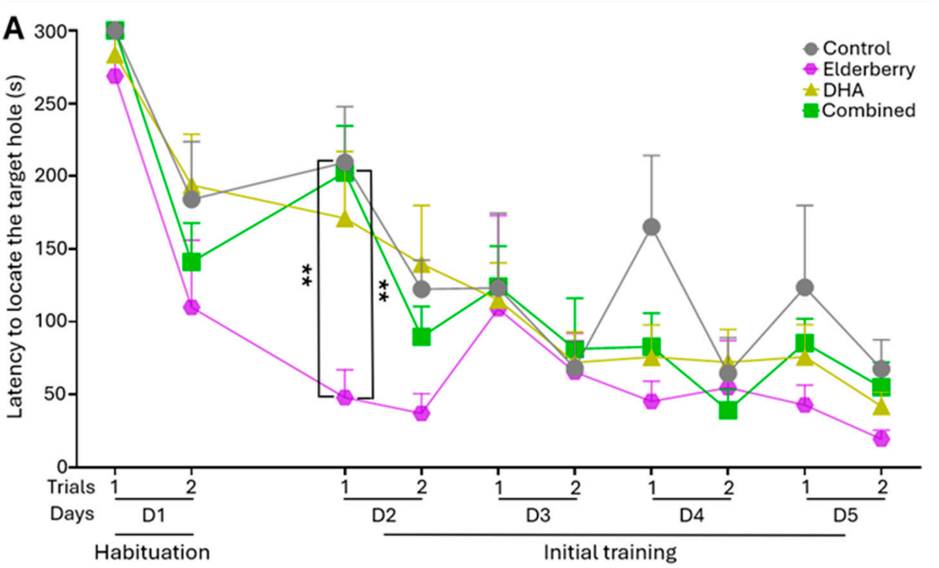


**Figure S3.** Barnes maze latency during habituation and initial acquisition training. Latency to enter the target hole during two daily trials on Day 1 (habituation) and Days 2–5 (initial training). Phase non-completers, defined as mice that failed to enter the target hole within 300 s in both trials on one or more acquisition days, were excluded from the acquisition-latency analysis. Mice with one unsuccessful trial on a given day were retained with the maximum latency of 300 s assigned for that trial. Data are presented as mean ± SEM and were analyzed using two-way repeated-measures ANOVA followed by Tukey's multiple-comparisons test. On Day 2, Trial 1, the elderberry group showed a significantly shorter latency than the control or combined groups, with $p < 0.01$ for both comparisons. No consistent group differences were observed during subsequent acquisition trials.

**Table S1.** Top-ranked GO functional-enrichment terms for proteins meeting the exploratory differential-abundance criterion in the elderberry, DHA, and combined groups relative to control.

| Comparison | Category | GO ID | GO Term | Count | Gene ratio | Raw p-value | FDR (q-value) |
|---|---|---|---|---|---|---|---|
| Elderberry vs. Control | Biological processes | GO:0061024 | membrane organization | 25 | 9.77% | 1.56E-06 | 3.15E-02 |
| | | GO:0007005 | mitochondrion organization | 17 | 6.64% | 2.88E-06 | 3.15E-02 |
| | | GO:0000209 | protein polyubiquitination | 13 | 5.08% | 5.70E-05 | 1.46E-01 |
| | | GO:0051224 | negative regulation of protein transport | 9 | 3.52% | 6.87E-05 | 1.46E-01 |
| | | GO:0050769 | positive regulation of neurogenesis | 13 | 5.08% | 8.70E-05 | 1.46E-01 |
| | Cellular components | GO:0098858 | actin-based cell projection | 13 | 5.08% | 8.70E-05 | 1.46E-01 |
| | | GO:0140168 | nuclear ribonucleoprotein granule | 18 | 7.03% | 1.02E-04 | 1.46E-01 |
| | | GO:0016607 | nuclear speck | 16 | 6.25% | 1.41E-04 | 1.54E-01 |
| | | GO:0071006 | U2-type catalytic step 1 spliceosome | 6 | 2.34% | 1.67E-04 | 1.55E-01 |
| | | GO:0071012 | catalytic step 1 spliceosome | 6 | 2.34% | 1.67E-04 | 1.55E-01 |
| | Molecular functions | GO:0016746 | acyltransferase activity | 21 | 8.20% | 1.24E-04 | 1.51E-01 |
| | | GO:0043021 | ribonucleoprotein complex binding | 10 | 3.91% | 1.33E-04 | 1.53E-01 |
| | | GO:0098847 | sequence-specific single stranded DNA binding | 3 | 1.17% | 6.18E-04 | 2.69E-01 |
| | | GO:0008320 | transmembrane protein transporter activity | 3 | 1.17% | 1.48E-03 | 3.73E-01 |
| | | GO:0015036 | disulfide oxidoreductase activity | 4 | 1.56% | 1.62E-03 | 3.93E-01 |
| DHA vs. Control | Biological processes | GO:0030029 | actin filament-based process | 94 | 6.53% | 6.02E-15 | 1.69E-12 |
| | | GO:0030036 | actin cytoskeleton organization | 87 | 6.05% | 1.08E-14 | 2.96E-12 |
| | | GO:0097435 | supramolecular fiber organization | 85 | 5.91% | 1.33E-12 | 2.45E-10 |
| | | GO:0007015 | actin filament organization | 51 | 3.54% | 5.72E-11 | 7.68E-09 |
| | | GO:0048858 | cell projection morphogenesis | 101 | 7.02% | 1.71E-20 | 1.34E-17 |
| | Cellular components | GO:0005884 | actin filament | 33 | 2.29% | 2.01E-11 | 2.91E-09 |
| | | GO:0044309 | neuron spine | 58 | 4.03% | 7.14E-18 | 3.07E-15 |
| | | GO:0043197 | dendritic spine | 56 | 3.89% | 5.43E-17 | 1.98E-14 |
| | | GO:0043198 | dendritic shaft | 21 | 1.46% | 8.51E-09 | 7.57E-07 |
| | | GO:0098982 | GABA-ergic synapse | 48 | 3.34% | 2.68E-17 | 1.01E-14 |
| | Molecular functions | GO:0003779 | actin binding | 79 | 5.49% | 6.12E-18 | 2.68E-15 |
| | | GO:0051015 | actin filament binding | 44 | 3.06% | 3.91E-11 | 5.49E-09 |
| | | GO:0015631 | tubulin binding | 66 | 4.59% | 1.79E-12 | 3.21E-10 |
| | | GO:0008017 | microtubule binding | 49 | 3.41% | 3.06E-10 | 3.54E-08 |
| | | GO:0003735 | structural constituent of ribosome | 30 | 2.08% | 2.77E-05 | 9.64E-04 |
| Combined vs. Control | Biological processes | GO:0006412 | translation | 23 | 5.34% | 2.89E-05 | 6.34E-02 |
| | | GO:0160307 | protein biosynthetic process | 23 | 5.34% | 2.89E-05 | 6.34E-02 |
| | | GO:0032543 | mitochondrial translation | 9 | 2.09% | 2.37E-04 | 1.80E-01 |
| | | GO:0140053 | mitochondrial gene expression | 10 | 2.32% | 5.11E-04 | 2.37E-01 |
| | | GO:0010256 | endomembrane system organization | 26 | 6.03% | 2.34E-05 | 6.34E-02 |
| | Cellular components | GO:0044391 | ribosomal subunit | 13 | 3.02% | 7.25E-05 | 1.03E-01 |
| | | GO:0015934 | large ribosomal subunit | 10 | 2.32% | 9.42E-05 | 1.21E-01 |
| | | GO:0000313 | organellar ribosome | 8 | 1.86% | 2.63E-04 | 1.86E-01 |
| | | GO:0005761 | mitochondrial ribosome | 8 | 1.86% | 2.63E-04 | 1.86E-01 |
| | | GO:0000315 | organellar large ribosomal subunit | 6 | 1.39% | 5.41E-04 | 2.37E-01 |
| | Molecular functions | GO:0003735 | structural constituent of ribosome | 11 | 2.55% | 2.45E-03 | 4.43E-01 |
| | | GO:0016301 | kinase activity | 25 | 5.80% | 5.32E-03 | 6.12E-01 |
| | | GO:0016247 | channel regulator activity | 10 | 2.32% | 4.20E-03 | 5.66E-01 |
| | | GO:0141108 | transporter regulator activity | 10 | 2.32% | 6.62E-03 | 6.83E-01 |
| | | GO:0019107 | myristoyltransferase activity | 3 | 0.70% | 1.39E-03 | 3.14E-01 |
